# Damage Evolution During Thread Rolling of TC16 Titanium Alloy Using Coupled Johnson-Cook Constitutive and Damage Models

**DOI:** 10.3390/ma19183942

**Published:** 2026-09-17

**Authors:** Jianxin Cao, Xin Song, Ning Han, Huiping Qi

**Affiliations:** School of Materials Science and Engineering, Taiyuan University of Science and Technology, Taiyuan 030024, China

**Keywords:** TC16 titanium alloy, Johnson-Cook constitutive model, thread rolling, damage model

## Abstract

In this work, a coupled Johnson-Cook (J-C) constitutive and damage modeling framework was established to investigate damage evolution during thread-rolling of TC16 titanium alloy. The J-C constitutive parameters were calibrated through tensile tests under different strain rates and temperatures, while the stress-triaxiality-dependent damage parameters were identified using specimens with different stress states. A finite element model of two-die radial thread rolling for MJ6 × 1 threads was developed using ABAQUS/Explicit. The mechanical response of the model was evaluated by comparing the numerically predicted and experimentally measured fracture displacements, with a maximum relative deviation of 7.63% in fracture displacement. The calibrated framework was subsequently applied to analyze stress distribution, plastic deformation localization, and damage evolution during the thread rolling process. The results indicate that damage accumulation is mainly concentrated at the thread root and the transition region between the thread flank and root. The damage of the thread can be attributed to the combined effects of high equivalent plastic strain, stress concentration, and an unfavorable stress state. The predicted damage localization agrees well with the experimentally observed fracture region under the investigated rolling condition. These findings demonstrate the applicability and limitations of the coupled Johnson-Cook constitutive damage framework for analyzing defect evolution in complex forming processes.

## 1. Introduction

In recent years, the demand for high-performance fasteners in the aerospace field has increased significantly due to the requirements for lightweight design, structural reliability, and long-term service performance [1,2]. Thread rolling is widely employed for manufacturing high-strength threaded components because it can improve surface integrity and fatigue performance compared with conventional machining processes [3,4]. Because of the stringent mechanical performance requirements for aerospace applications, titanium alloys are widely used for manufacturing aerospace fasteners. TC16 titanium alloy is a typical α + β titanium alloy used in aerospace applications because of its high specific strength and favorable mechanical properties [5]. However, the poor room-temperature formability of the titanium alloy may cause different types of damage during the forming process [6,7].

During thread rolling, the material undergoes severe plastic deformation under non-uniform loading conditions. The interaction between geometric constraints, stress concentration, strain localization, and temperature-dependent material behavior makes it difficult to experimentally characterize the internal stress and damage evolution processes. Finite element simulation has therefore become an important tool for understanding and optimizing complex forming processes. The accuracy of such simulations strongly depends on the reliability of the constitutive and damage models used to describe material behavior. Among various constitutive models, the Johnson-Cook (J-C) model is widely used in engineering simulations involving large deformation because it separately considers strain hardening, strain-rate sensitivity, and thermal softening effects [8,9]. Its relatively simple mathematical form and experimentally identifiable parameters make it suitable for industrial-scale forming simulations.

However, the J-C model is a commonly used constitutive model for metallic materials and does not explicitly consider microstructural mechanisms such as crystallographic texture evolution, phase transformation, or grain-scale damage development. Therefore, although it may not fully describe the underlying physical mechanisms of titanium alloy deformation, it provides an effective engineering approximation for predicting macroscopic mechanical responses under complex thermo-mechanical loading conditions. In the present study, the J-C model was selected because thread rolling mainly involves large plastic deformation, strain-rate effects, and temperature variation, which correspond well with the dominant factors considered by the model. In addition to describing plastic deformation behavior, the accurate prediction of failure during forming processes requires an appropriate damage model. The J–C damage model extends the J-C framework by introducing the effects of stress triaxiality, strain rate, and temperature on fracture strain through a cumulative damage formulation [10,11]. This makes it suitable for analyzing ductile damage evolution under complex loading paths. Nevertheless, the reliability of the J-C damage model depends strongly on the calibration methodology and experimental characterization of different stress states.

Previous studies have investigated the constitutive response and damage behavior of TC16 titanium alloy under different deformation conditions, including Johnson-Cook-based and other constitutive descriptions of its mechanical response [12,13]. These studies provide an important basis for describing the macroscopic flow behavior of TC16 titanium alloy. In addition, stress-state-dependent ductile damage during cold upsetting of TC16 fasteners has been investigated, demonstrating the important influence of multiaxial stress states on damage evolution. However, thread rolling differs substantially from conventional tensile and upsetting deformation because the material experiences repeated contact loading, highly localized material flow, steep strain gradients, and continuously evolving stress states near the thread root. Consequently, the relationship between local stress state, accumulated plastic deformation, and progressive damage localization during TC16 thread rolling remains insufficiently understood.

Accordingly, the objective of the present study is to investigate the evolution and localization of ductile damage during thread rolling of TC16 titanium alloy using a coupled Johnson-Cook constitutive and damage framework. Particular attention is given to the relationship among the local stress state, accumulated plastic strain, and the development of the critical damage region at the thread root. By comparing the predicted damage localization with experimentally observed fracture locations, this work aims to clarify the mechanical conditions responsible for thread-root damage and to provide a numerical basis for subsequent optimization of TC16 thread rolling processes.

## 2. Materials and Methods

### 2.1. Materials and Mechanical Experiments

The material used in this work is the commercial TC16 titanium alloy. The chemical composition of the alloy is shown in Table 1. The chemical composition was obtained from the material supplier’s certificate (Baoji Titanium Industry Co., Ltd., Baoji, China) and verified by inductively coupled plasma optical emission spectroscopy (ICP-OES) (Thermo Scientific™ iCAP™ PRO XP, Thermo Fisher, Waltham, MA, USA).

To characterize the deformation behavior of TC16 titanium alloy under different conditions, the tensile experiments were conducted using a Gleeble-3500 thermo-mechanical simulator (Dynamic Systems Inc. (DSI), Poestenkill, NY, USA). Each mechanical test was repeated three times under identical conditions. The experimental results were reported as mean values, and the standard deviations were calculated to evaluate experimental repeatability. The tests were performed under different strain rates and temperatures to identify the parameters associated with strain hardening, strain-rate sensitivity, and thermal softening in the Johnson-Cook constitutive model. The mechanical test parameters are shown in Table 2. The averaged stress–strain responses were subsequently used for calibration of the Johnson-Cook constitutive parameters.

**Figure 1:**
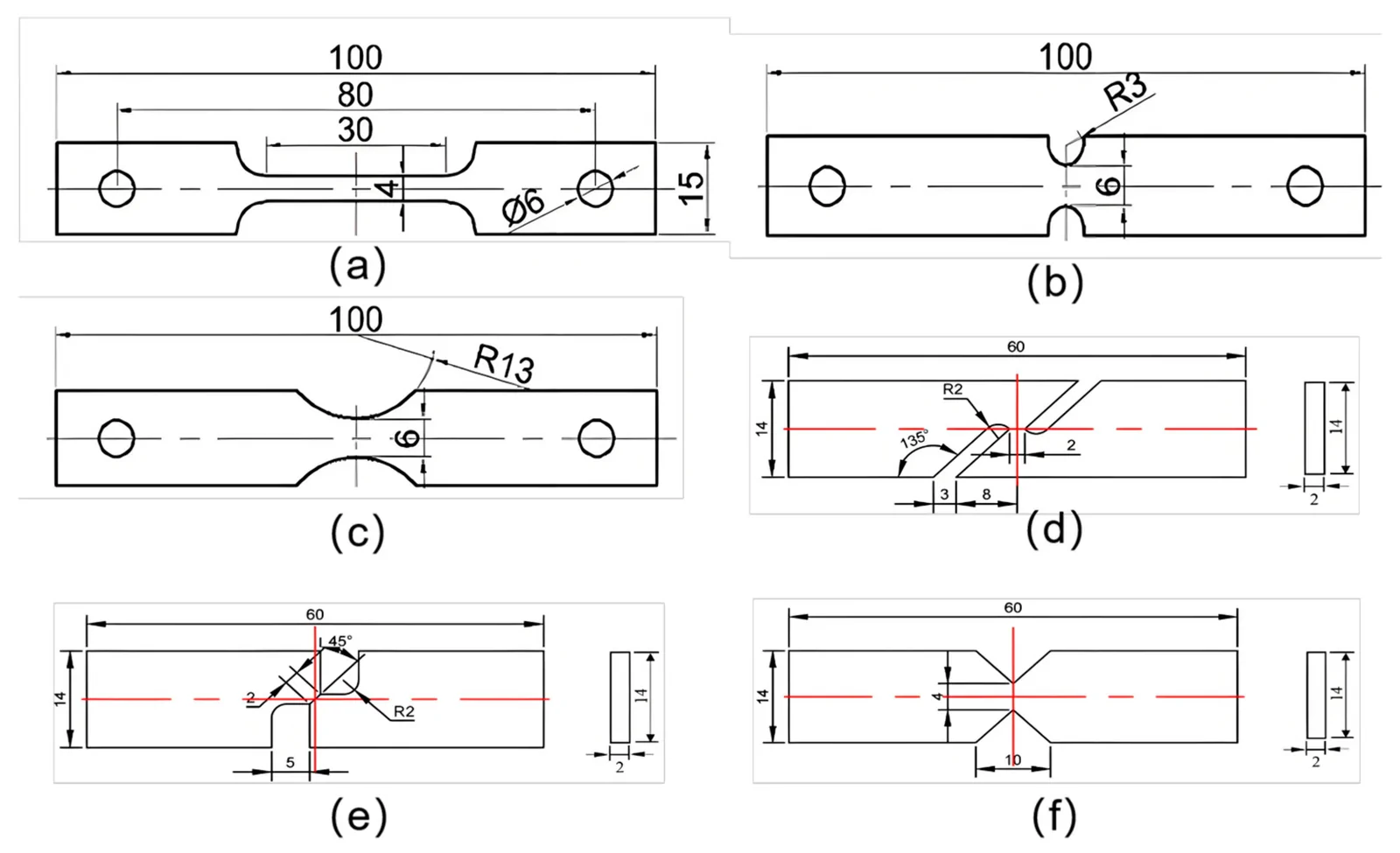
The size of the mechanical test samples: (**a**) Smooth tensile sample, (**b**) the notched R3 sample, (**c**) the notched R13 sample, (**d**) the 0° shear sample, (**e**) the 45° shear sample, (**f**) the 90° shear sample [14].

The thread rolling process involves complex stress states ranging from tensile-dominated loading to shear-dominated deformation. Therefore, different specimen geometries were designed for damage model calibration, including smooth tensile specimens, notched tensile specimens, and shear specimens. The smooth tensile specimens were used to characterize the fracture behavior under relatively low stress triaxiality conditions. The notched specimens with radii of R = 3 mm and R = 13 mm were selected to introduce different levels of stress triaxiality through geometric constraint. The shear specimens with different shear angles were employed to obtain deformation states dominated by shear loading. These specimens together provide a relatively wide range of stress states required for calibrating the stress-triaxiality-dependent Johnson-Cook damage model. The samples were machined using electric discharge wire cutting according to the dimensions shown in Figure 1.

### 2.2. Johnson-Cook Constitutive Model

The J-C constitutive model is employed to describe the plastic flow behavior of TC16 titanium alloy. The model expresses the von Mises flow stress as a product of three independent functions, accounting for strain hardening, strain-rate hardening, and thermal softening [15]:

(1) $$\bar{\sigma }\;=\;\left(A\;+\;B\varepsilon^{n}\right)\left(1\;+\;Cln\frac{\dot{\varepsilon }}{{\dot{\varepsilon }}_{0}}\right)\;\times \;\left[1\;-\;{\left(\frac{T-T_{r}}{T_{m}-T_{r}}\right)}^{m}\right]$$

where $\bar{\sigma }$ is the von Mises stress, *ε* is the equivalent plastic strain, $\dot{\varepsilon }$ is the plastic strain rate, ${\dot{\varepsilon }}_{0}$ is the reference strain rate (taken as 0.001 s^−1^), *T* is the current temperature, $T_{r}$ is the reference temperature (20 °C), and $T_{m}$ is the melting temperature of the alloy (1650 °C). The five material constants are A (initial yield stress at reference conditions), B (strain hardening modulus), *n* (strain hardening exponent), *C* (strain-rate sensitivity coefficient), and *m* (thermal softening exponent). These parameters are to be determined experimentally via the decoupled calibration method.

To predict fracture initiation and evolution during thread rolling, the J-C damage model is adopted. The model defines the equivalent plastic strain at fracture ${\bar{\epsilon }}_{f}^{pl}$ as a function of stress triaxiality, strain rate, and temperature:

(2) $$\begin{array}{c} {\bar{\epsilon }}_{f}^{pl}\;=\;\left[d_{1}\;+\;d_{2}\exp\left(d_{3}\eta \right)\right]\left[1\;+\;d_{4}\ln\left(\frac{{\dot{\bar{\epsilon }}}^{pl}}{{\dot{\epsilon }}_{0}}\right)\right]\left[1\;+\;d_{5}T^{*}\right] \end{array}$$

where $\eta \;=\;\sigma_{m}/\bar{\sigma }$ is the stress triaxiality and $\sigma_{m}$ is the hydrostatic stress. $T^{*}\;=\;(T\;-\;Tr)/(Tm\;-\;Tr)$ is the homologous temperature. The parameters *d*_1_~*d*_5_ are fitting coefficients to be calibrated. Parameters *d*_1_~*d*_3_ describe the dependence of fracture strain on stress triaxiality, *d*_4_ characterizes the strain-rate sensitivity of fracture, and *d*_5_ represents the temperature sensitivity. Damage accumulation in the material follows a linear cumulative law. The scalar damage value *D* is defined as

(3) $$\begin{array}{c} D\;=\;\sum \frac{\Delta {\bar{\epsilon }}^{pl}}{{\bar{\epsilon }}_{f}^{pl}}\;=\;\int_{0}^{{\bar{\epsilon }}^{pl}}\frac{d{\bar{\epsilon }}^{pl}}{{\bar{\epsilon }}_{f}^{pl}\left(\eta ,{\dot{\bar{\epsilon }}}^{pl},T\right)} \end{array}$$

where $\Delta {\bar{\epsilon }}^{pl}$ is the increment of equivalent plastic strain at each integration step. The damage value D ranges from 0 (undamaged state) to 1 (numerical failure threshold). When D reaches unity at an integration point, the corresponding finite element is deleted to simulate macroscopic crack propagation.

### 2.3. Finite Element Modeling

**Figure 2:**
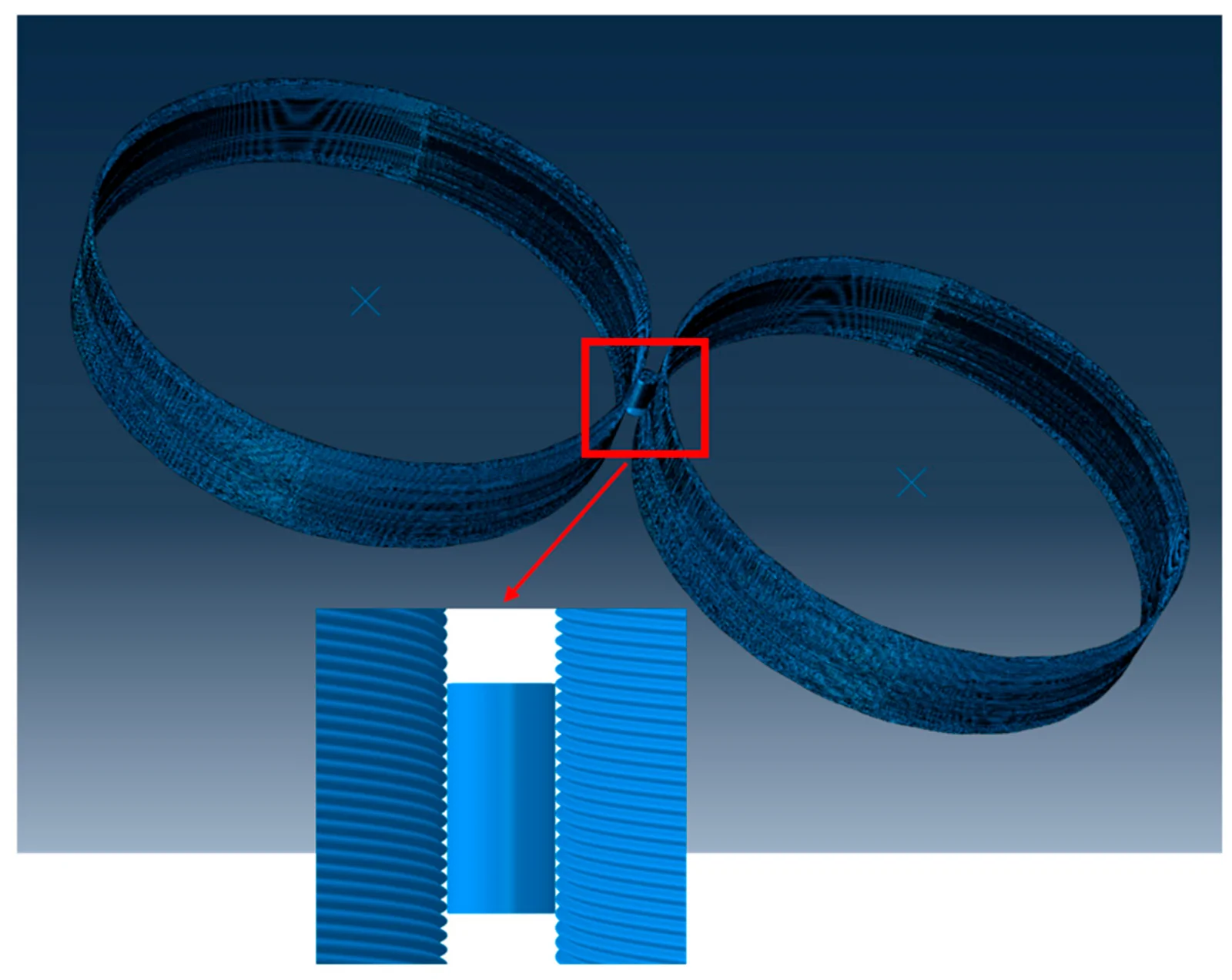
The thread rolling finite element simulation model.

To evaluate the applicability of the proposed constitutive and damage models, finite element (FE) simulations were performed for both the mechanical tests and the thread rolling process using ABAQUS/Explicit 2021. A three-dimensional thermo-mechanically coupled FE model of the two-die radial thread rolling process for an MJ6 × 1 external thread was established, and the corresponding process parameters are summarized in Table 3. The finite element model developed in this paper is shown in Figure 2. A surface-to-surface contact formulation was defined between the rolling dies and the workpiece. The normal contact behavior was modeled using hard contact, while the tangential behavior was described using a Coulomb friction model with a friction coefficient of 0.15.

The workpiece was discretized using eight-node thermally coupled elements with reduced integration (C3D8RT). This element formulation was selected because it efficiently captures the coupled thermo-mechanical behavior associated with large plastic deformation, strain-rate effects, and friction-induced heat generation during thread rolling, while maintaining good computational efficiency for explicit dynamic analysis. The rolling dies were modeled as rigid bodies since their elastic deformation is negligible compared with that of the workpiece. The workpiece mesh consisted of approximately 180,000 elements. A global mesh size of 0.5 mm was adopted, while local mesh refinement was applied to the contact region and the thread-root area, where severe plastic deformation and damage localization are expected. The minimum element size in the refined region was 0.03 mm. A mesh sensitivity study was performed using coarse, medium, and fine meshes. The difference in the predicted maximum damage value and equivalent plastic strain between the medium and fine meshes was less than 5%, confirming mesh-independent predictions. Furthermore, all three mesh densities predicted the maximum damage region at the thread-root transition area, indicating that the predicted damage localization was insensitive to mesh refinement.

To avoid excessive mesh distortion caused by large material flow during thread rolling, the Arbitrary Lagrangian–Eulerian (ALE) adaptive meshing technique was employed. In ABAQUS/Explicit, adaptive meshing was performed with an adaptive-mesh frequency of 10 and 3 mesh sweeps per adaptive meshing increment. During the ALE remeshing process, all relevant solution variables, including equivalent plastic strain, temperature, and damage-related state variables, were transferred to the updated mesh to preserve the deformation history throughout the simulation. A mass scaling factor of 50 was applied to improve computational efficiency. The influence of mass scaling was evaluated by monitoring the ratio of kinetic energy to internal energy throughout the simulation. The kinetic energy remained below 5% of the internal energy during the entire rolling process and below 3% during the stable rolling stage, indicating that inertial effects introduced by mass scaling were negligible. Therefore, the adopted mass scaling strategy was considered acceptable for the present explicit simulation.

## 3. Results and Discussion

### 3.1. Results of Mechanics Experiments

**Figure 3:**
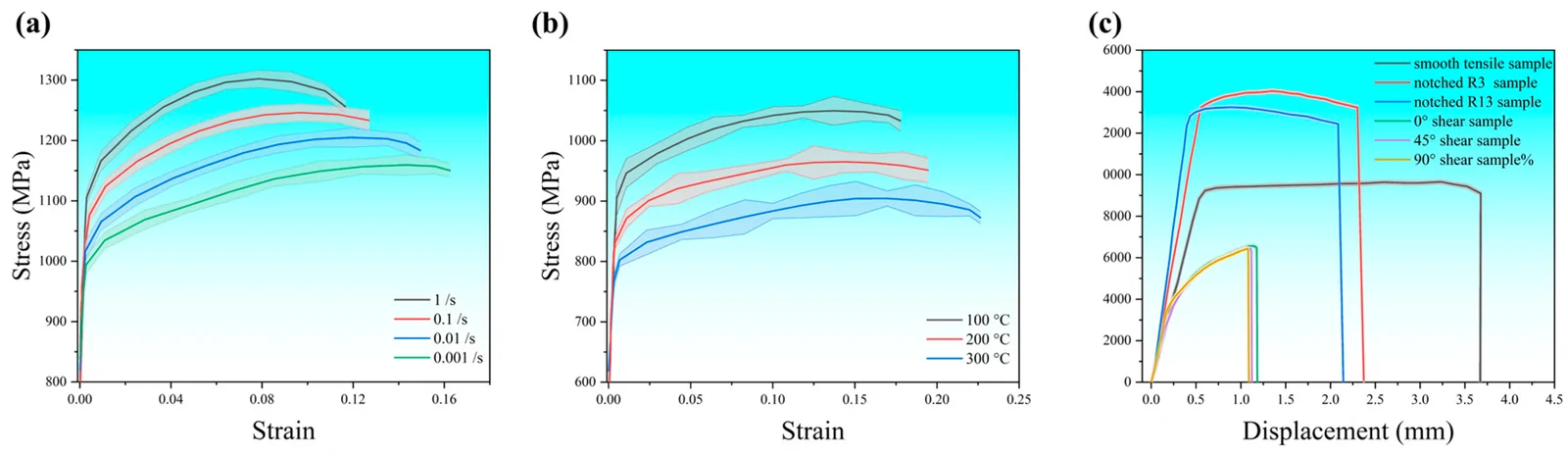
The mechanical test results of different samples: (**a**) the samples with different strain rates, (**b**) the samples with different temperatures, (**c**) the samples with different shapes.

The mechanical test results are shown in Figure 3. The solid lines represent the average responses obtained from three repeated experiments, and the shaded regions indicate the corresponding standard deviations. The stress–strain curves obtained from the smooth tensile samples at various strain rates (20 °C) and various temperatures (0.001 s^−1^) show typical work-hardening behavior followed by necking. The material exhibits clear strain-rate sensitivity. Increasing the strain rate from 0.001 s^−1^ to 1 s^−1^, the flow stress rises from 1120 MPa to 1250 MPa. It also shows a significant thermal softening phenomenon. The flow stress decreases from 1050 MPa to 870 MPa when increasing the temperature from 100 °C to 300 °C. For the damage model calibration, the fracture displacements and loads of the six sample geometries were recorded. The smooth tensile sample exhibits the largest fracture displacement (3.68 mm), while the notched samples show earlier fracture due to stress concentration. The shear samples exhibit the smallest displacements and lower peak loads. These data serve as the basis for extracting the fracture strain and average stress triaxiality for each geometry in the following parameter calculation.

### 3.2. Calculation of Parameters for the J-C Constitutive Model

At reference conditions (20 °C, 0.001 s^−1^), Equation (1) reduces to

(4) $$\begin{array}{c} \sigma \;=\;A\;+\;B\varepsilon^{n} \end{array}$$

The initial yield stress A = 1025 MPa is obtained directly from the 0.2% offset yield stress of the experimental curve. Subtracting *A* from the flow stress data for ε > 0.002 and taking natural logarithms transforms this to

(5) $$\begin{array}{c} \ln\left(\sigma \;-\;A\right)=\ln B\;+\;\mathrm{nln}\varepsilon \end{array}$$

**Figure 4:**
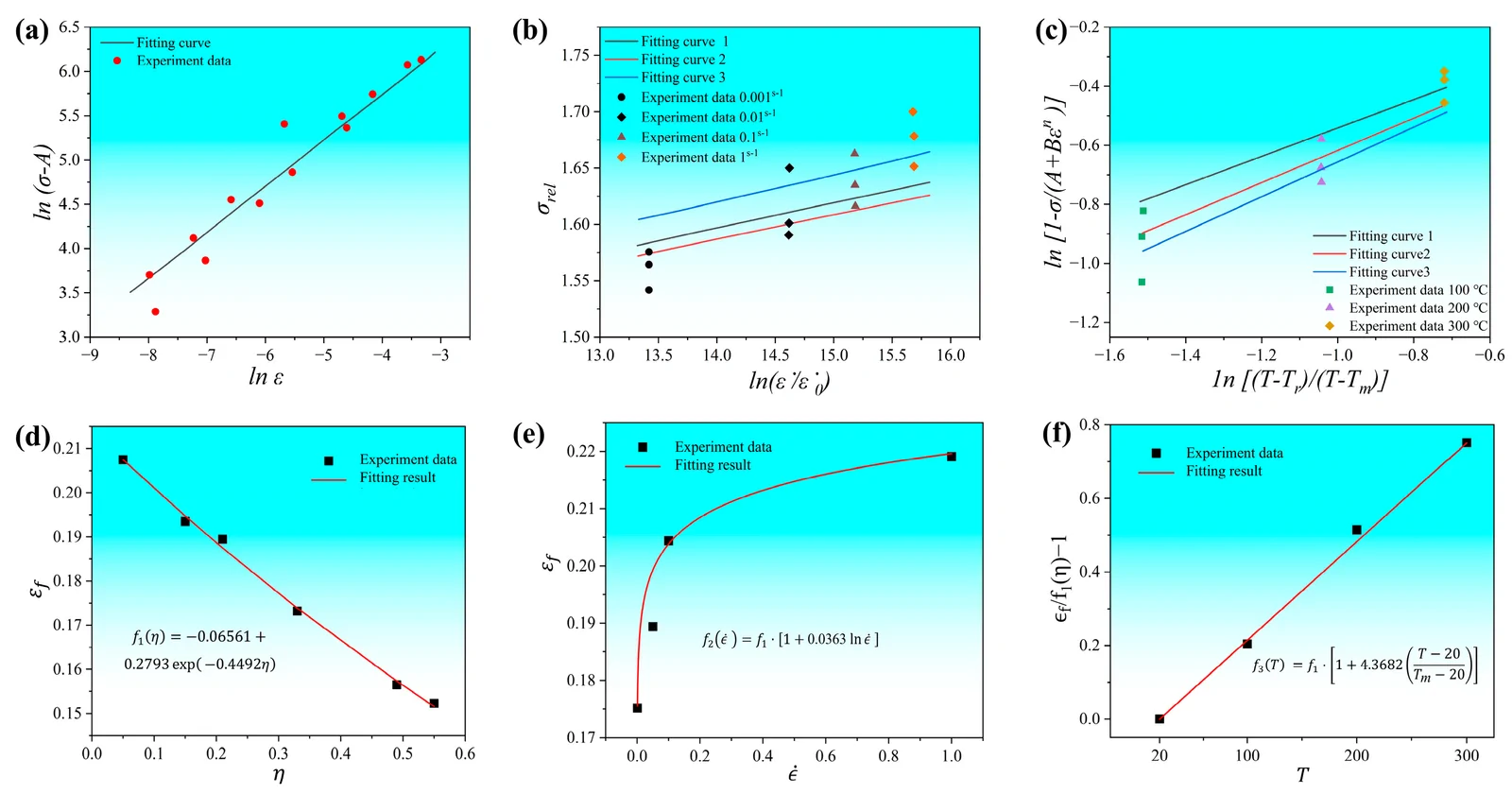
Calibration of the Johnson-Cook constitutive and damage parameters: (**a**) $ln\left(\sigma -A\right)$-lnε, (**b**) $\sigma_{rel}$-$ln\frac{\dot{\varepsilon }}{{\dot{\varepsilon }}_{0}}$, (**c**) $ln\;[1-\sigma /(A+B\varepsilon^{n})]$*-*$ln\;[(T-T_{r})/(T-T_{m})]$, (**d**) $\varepsilon_{f}$-η, (**e**) $\varepsilon_{f}$-$\dot{\varepsilon }$, (**f**) $\frac{\varepsilon_{f}}{f_{1}\left(\eta \right)}-1$-*T*.

By plotting *ln* (*σ* − *A*) against *lnε* and applying linear least-squares regression (Figure 4a), the slope gives *n* = 0.42, and the intercept gives *B* = 384 MPa. To determine the strain-rate sensitivity coefficient C, Equation (1) at the reference temperature can be rewritten as

(6) $$\sigma \;=\;\left(A\;+\;B\varepsilon^{n}\right)\left(1\;+\;\mathrm{Cln}\frac{\dot{\varepsilon }}{{\dot{\varepsilon }}_{0}}\right)$$

Using fixed plastic strain levels of 0.08, 0.10, and 0.12, the flow stresses at 0.001, 0.01, 0.1, and 1 s^−1^ were extracted (Table 4). The relative stress is defined as *σ_rel_* = *σ*($\dot{\varepsilon }$)/*σ*_0_; *σ_rel_* can be written as

(7) $$\begin{array}{c} \sigma_{rel}\;=\;1\;+\;\mathrm{Cln}\frac{\dot{\varepsilon }}{{\dot{\varepsilon }}_{0}} \end{array}$$

$\sigma_{\mathrm{rel}}$ is plotted against *ln*($\dot{\varepsilon }$/${\dot{\varepsilon }}_{0}$) data and a linear regression is conducted (Figure 4b). The slope obtained by linear regression gives *C* = 0.0261. The stress under different strain rates is extracted and shown in Table 4.

To determine the thermal softening exponent *m*, Equation (1) at the reference strain rate gives

(8) $$\sigma \;=\;\left(A\;+\;B\varepsilon^{n}\right)\left[1-{\left(\frac{T-T_{r}}{T_{m}-T_{r}}\right)}^{m}\right]$$

Using the flow stresses at 100, 200, and 300 °C at fixed strain levels (Table 5), the normalized flow stress values were plotted against $lnT^{*}$ and linear regression was performed (Figure 4c), and *m* = 0.8027. All constitutive parameters are summarized in Table 6.

To calibrate the stress triaxiality parameters (*d*_1_, *d*_2_, *d*_3_), the fracture strain *ε_f_* and the average stress triaxiality $\bar{\eta }$ must be determined for each of the six sample geometries. Since stress triaxiality evolves during deformation, $\bar{\eta }$ is calculated from finite element simulations of each tensile test using the integral

(9) $$\begin{array}{c} \bar{\eta }\;=\;\frac{1}{\epsilon_{f}}\int_{0}^{\epsilon_{f}}\eta (\epsilon_{pl})d\epsilon_{pl} \end{array}$$

The experimentally measured fracture strains and the corresponding $\bar{\eta }$ values are listed in Table 7. It should be noted that fracture displacement is not a direct indicator of stress triaxiality. Although the R = 13 mm specimen exhibits a smaller fracture displacement than the R = 3 mm specimen, the higher average stress triaxiality of the R = 3 mm specimen is attributed to its stronger geometric constraint and larger stress concentration effect.

**Figure 5:**
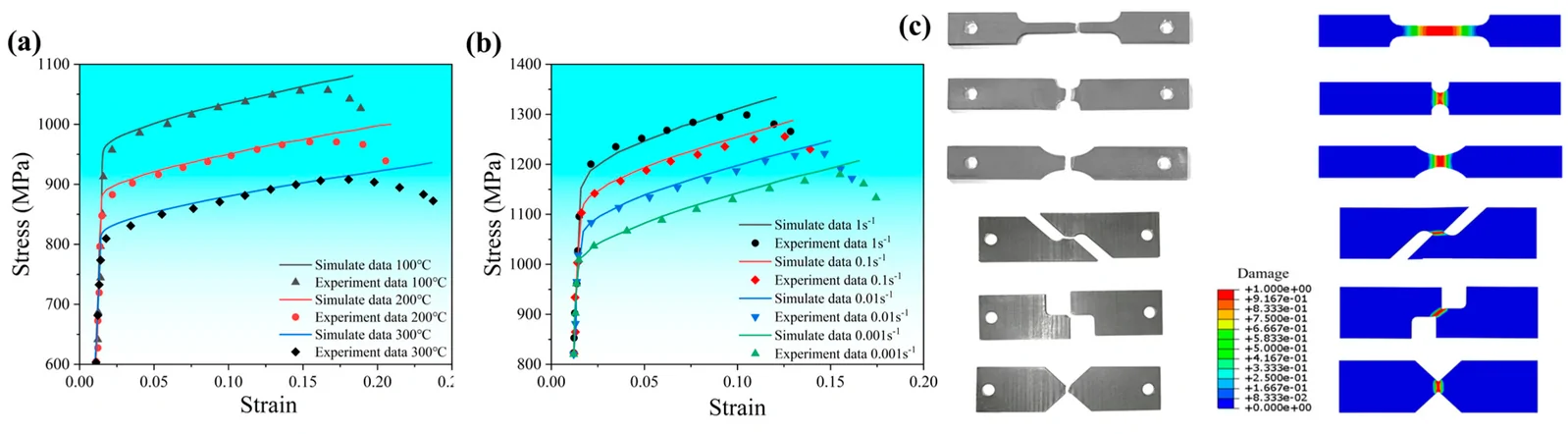
Comparison between finite element predictions and experimental results: (**a**) the samples under different temperatures, (**b**) the samples under different strain rates, (**c**) the comparison of fracture morphology and damage field distribution of samples.

Since the average stress triaxiality cannot be directly measured experimentally, it was extracted from finite element simulations of the corresponding mechanical tests. Before being used for damage parameter calibration, the FE models were validated by comparing the predicted stress–strain responses with the experimental results (Figure 5a,b). The reasonable agreement between simulations and experiments provides confidence that the calculated stress triaxiality histories are sufficiently reliable for calibrating the Johnson-Cook damage model.

Using the six data points ($\varepsilon_{f}$, $\bar{\eta }$) and the reduced form of Equation (2) at the reference strain rate and temperature,

(10) $${\bar{\epsilon }}_{f}^{pl}\;=\;d_{1}\;+\;d_{2}\exp\left(d_{3}\eta \right)$$

A nonlinear least-squares fit was performed using the Levenberg–Marquardt algorithm (Figure 4d). The resulting parameters are *d*_1_ = −0.066, *d*_2_ = 0.279, *d*_3_ = −0.449.

The strain-rate parameter *d*_4_ was determined from tests on smooth samples at various strain rates (0.001, 0.01, 0.1, and 1 s^−1^) at room temperature. Using the already-determined stress triaxiality term *f*_1_(*η*), Equation (2) reduces to

(11) $$\begin{array}{c} \frac{{\bar{\epsilon }}_{f}^{pl}\left({\dot{\epsilon }}^{*}\right)}{f_{1}}\;-\;1\;=\;d_{4}\ln\left({\dot{\epsilon }}^{*}\right) \end{array}$$

Linear regression of this relationship gives *d*_4_ = 0.0363. The temperature parameter d5 was determined from tests on smooth samples at 100, 200, and 300 °C at the reference strain rate. Using the same reduction,

(12) $$\begin{array}{c} \frac{{\bar{\epsilon }}_{f}^{pl}\left(T^{*}\right)}{f_{1}}\;-\;1\;=\;d_{5}T^{*} \end{array}$$

Linear regression gives *d*_5_ = 4.37. The nonlinear fitting of Equation (10) yielded a coefficient of determination(*R*^2^) greater than 0.98. Substituting all calibrated values into Equation (2), the complete J-C damage model for TC16 titanium alloy is

(13) $${\bar{\epsilon }}_{f}^{pl}\;=\;\left[-0.066\;+\;0.279\exp\left(-0.449\eta \right)\right]\cdot \left[1\;+\;0.036\ln\left(\frac{{\dot{\bar{\epsilon }}}^{pl}}{{\dot{\epsilon }}_{0}}\right)\right]\cdot \left[1+4.37\;T^{*}\right]$$

### 3.3. The Validation of Damage Models

The constitutive and damage models were calibrated using experiments covering strain rates from 0.001~1 s^−1^ and temperatures from 20~300 °C. The strain rate ranged from 0.01 to 0.73 s^−1^, the temperature ranged from 20 to 250 °C, and the stress triaxiality in the thread-root region ranged from −0.3 to 0.4. Although transient negative stress triaxiality occurred during local contact deformation, the region associated with maximum damage was mainly subjected to non-negative stress triaxiality. The stress triaxiality in the damage localization region was mainly within *η* ≈ 0~0.4, which is consistent with the experimental calibration range. The calibrated constitutive and damage models were first evaluated by comparing the simulated and experimental stress–strain responses under different temperatures and strain rates. As shown in Figure 5a,b, the simulated stress–strain responses reproduce the experimental trends under different temperatures and strain rates. The maximum relative deviation of fracture displacement between the numerical predictions and experimental measurements is 7.63%, as summarized in Table 8. In addition, the predicted fracture locations agree well with the experimentally observed fracture morphologies for all six specimen geometries. These results indicate that the proposed framework can reasonably reproduce the macroscopic deformation and damage localization under the investigated loading conditions.

Furthermore, the damage field distribution of different samples was extracted and compared with the macromorphology of samples. The predicted damage localization showed good consistency with the experimentally observed fracture locations in Figure 5c. The damage field distribution was mainly concentrated at the notch root for the notched samples and along the shear plane for the shear samples. Overall, the comparisons indicate that the calibrated J–C damage model can reasonably reproduce the deformation behavior and damage localization observed under the investigated loading conditions.

### 3.4. Damage Analysis of the Thread Rolling Process

**Figure 6:**
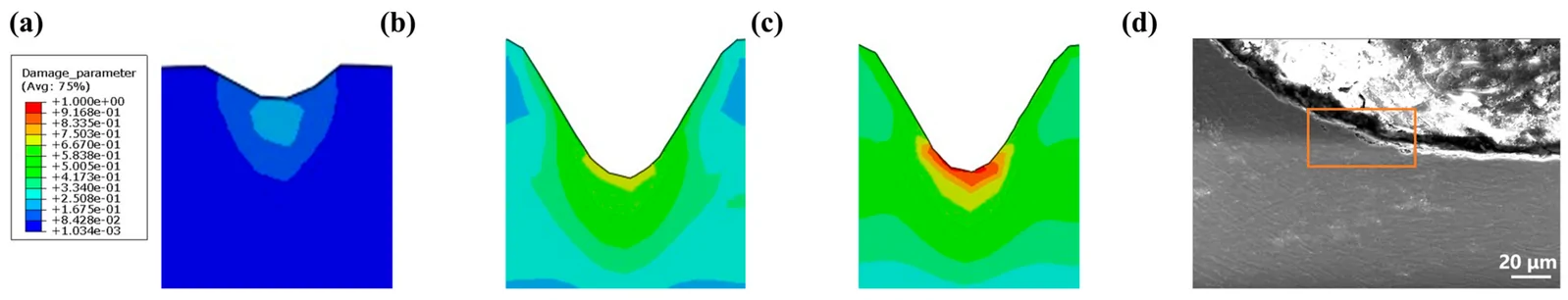
Damage evolution during thread rolling: (**a**) the biting stage, (**b**) the stable rolling stage, (**c**) the finishing stage, and (**d**) experimentally observed cracking at the thread-root region [14].

**Figure 7:**
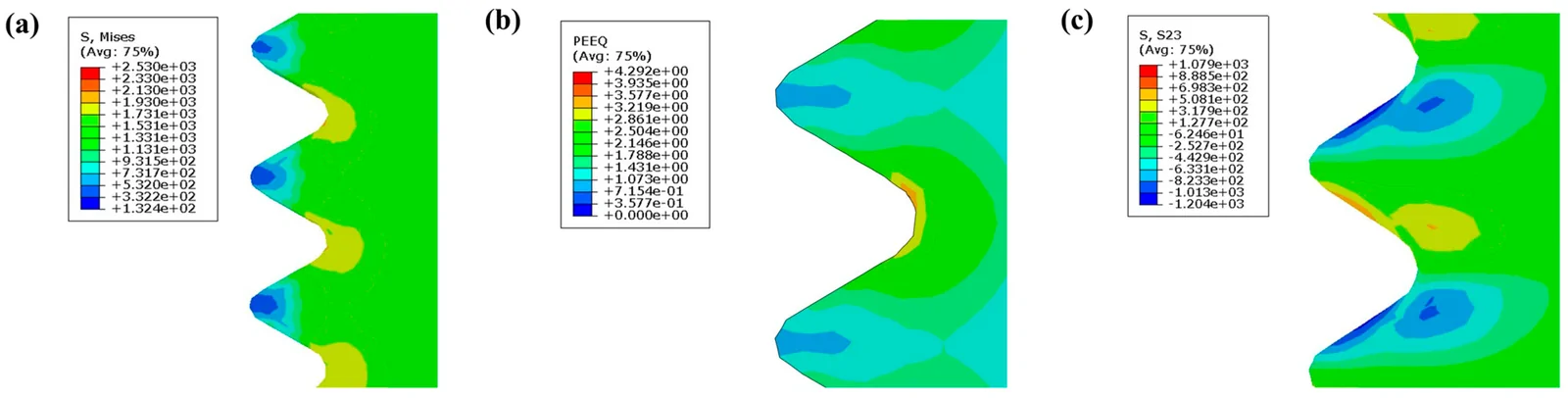
(**a**) The Mises stress distribution of the thread rolling process, (**b**) the PEEQ distribution of the thread rolling process, (**c**) the shear stress distribution of the thread rolling process [14].

In the present study, the damage variable *D* represents cumulative ductile damage. Values of 0 < *D* < 1 describe progressive damage accumulation within the Johnson-Cook framework. When *D* reaches 1 at an integration point, the numerical failure criterion is satisfied and element deletion is activated. Therefore, *D =* 1 is interpreted as the numerical failure threshold adopted in the finite element model rather than as a direct representation of physical crack initiation or the complete crack-propagation process. It should be noted that element deletion is a numerical treatment and does not represent the complete physical process of crack propagation. Figure 6 shows the damage field distribution during the thread rolling process. It can be observed that the highest damage values are concentrated at the thread root and the junction between the flank and the bottom of the thread. The other regions, such as the thread crest and flank, exhibit lower damage values. The Mises stress, equivalent plastic strain (PEEQ) and shear stress distribution shown in Figure 7 can help to understand the damage mechanism of thread rolling.

The evolution of damage during the thread rolling process is closely related to the radial feed motion of the rolling dies. When the rolling dies first contact the workpiece surface, the contact area is limited to small regions on the blank surface. In this stage, the material undergoes plastic deformation and the damage value *D* increases from 0 to 0.2. As the dies continue to feed, the contact pressure increases. The thread flank starts to form and the damage value of the thread-root region increases continuously. The damage value of the thread-root region increases to 0.75 and initial localization at the root transition region becomes evident. The damage is primarily concentrated at the surface layer of the root. When the dies approach their final radial position, the tooth profile is nearly fully formed. The material at the root is now severely squeezed between the die flank and the underlying substrate. The maximum PEEQ value occurs at the thread-root surface. At the same time, the damage area expands from the surface into the subsurface region, but the highest values remain concentrated at the root surface. During the final stage of rolling, the dies reach the maximum feed position to size the thread and improve surface finish. Although the additional plastic strain increment during this stage is small, the continuous deformation during the final rolling stage from successive die teeth causes further damage accumulation at the root. The damage value at the thread root gradually approaches the critical value. When *D* = 1, the numerical failure criterion is satisfied and local element deletion is activated. This is consistent with the damage observed in the actual samples (shown in Figure 6d). As the rolling process proceeds to the final stage, the damage value at the thread root eventually reaches *D* = 1, indicating that the numerical failure threshold has been reached in the thread-root region. The flank and crest, by contrast, exhibit lower *D* values than the thread-root region.

The preferential accumulation of damage at the thread-root transition region can be explained by the combined effects of contact mechanics and localized plastic deformation during thread rolling. The stress state at the thread root is characterized by high equivalent stress, elevated stress triaxiality, and significant shear deformation. During rolling, the dies apply both normal contact pressure and tangential friction stress to the workpiece surface, forcing the material to flow plastically into the thread cavity. Material flow is restricted by the geometric constraint at the thread root, resulting in localized stress concentration. In addition, the relatively small radius of curvature further amplifies the local equivalent stress. The local high von Mises stress at the root surface is significantly amplified relative to the nominal stress level [14]. The simulation shows that the Mises stress at the root surface far exceeds the values on the crest or flank. The elevated equivalent stress promotes localized plastic deformation and damage accumulation.

In addition to stress concentration, severe plastic strain localization occurs at the thread root. The rolling pressure forces the surface material to deform within a narrow region adjacent to the die corner, while the underlying material constrains the deformation. As a result, localized shearing develops at the thread root, as evidenced by the PEEQ distribution. The simulation shows that the equivalent plastic strain at the thread-root surface is significantly higher than that at the thread crest and flank. According to the calibrated stress triaxiality dependence of the Johnson-Cook damage model, an increase in positive stress triaxiality within the investigated range is associated with a reduction in the predicted fracture strain, thereby accelerating damage accumulation under continued plastic deformation [16,17,18,19]. Furthermore, the tangential friction stress acting along the die–workpiece interface contributes to the local shear deformation, while the normal contact pressure increases the stress triaxiality in the thread-root region [20,21,22]. The combined effects of high equivalent plastic strain, elevated stress triaxiality, and shear deformation make the thread root the most damage-prone region under the investigated rolling condition.

The finite element results indicate that the thread-root region experiences the highest equivalent plastic strain together with elevated stress triaxiality and shear stress. These conditions are generally considered favorable for ductile damage initiation in titanium alloys according to previous studies. However, the present simulations do not directly resolve microstructural fracture mechanisms. Previous studies have suggested that multiaxial stress states may promote micro-void nucleation at grain boundaries and α/β phase interfaces, which are common damage initiation sites in two-phase titanium alloys [23]. Likewise, the combined effects of high shear stress and hydrostatic tension have been reported to facilitate the transition from damage accumulation to macroscopic cracking. Therefore, these mechanisms should be regarded as possible explanations rather than conclusions directly demonstrated by the present simulations. The numerical results indicate that the surface layer at the thread root is subjected to the most severe stress and plastic deformation. The predicted damage localization is consistent with the regions of maximum equivalent stress and plastic strain. Furthermore, the steep strain gradient between the surface and the interior suggests pronounced deformation incompatibility at the thread-root transition, leading to localized shearing and accelerated damage accumulation.

In summary, the thread-root transition region is the most damage-prone location during thread rolling because of the combined effects of stress concentration, localized plastic deformation, elevated stress triaxiality, and stress-state-dependent damage evolution. The geometric constraint at the thread root restricts material flow and promotes localized strain accumulation, resulting in a steep strain gradient between the surface and the underlying material. Under continuous deformation during the rolling process, damage accumulates progressively at the thread root according to the calibrated J–C damage model, and the damage variable eventually reaches the numerical failure threshold under the investigated rolling condition. The above observations are valid for the investigated thread rolling condition. The influences of rolling speed, feed rate, lubrication condition, and thread geometry on damage evolution were not systematically investigated in the present study and therefore require further investigation. Nevertheless, the present results provide insight into the damage evolution and localization during TC16 titanium alloy thread rolling and may serve as a reference for future optimization of thread rolling processes and thread geometry.

## 4. Conclusions

In this study, a coupled Johnson-Cook constitutive and damage model framework was developed to investigate damage initiation during thread rolling of TC16 titanium alloy. The main conclusions obtained from the experimental characterization and finite element analysis are summarized as follows:

1. The J-C constitutive parameters of TC16 titanium alloy were determined as *A* = 1025 MPa, *B* = 384 MPa, *n* = 0.42, *C* = 0.0261, and *m* = 0.8027. The damage model parameters were obtained as *d*_1_ = −0.066, *d*_2_ = 0.279, *d*_3_ = −0.449, *d*_4_ = 0.036, and *d*_5_ = 4.37.
2. The mechanical response of the calibrated framework was evaluated by comparison with the experimental measurements. The maximum relative deviation between the numerically predicted and experimentally measured fracture displacements was 7.63%. This displacement-based deviation characterizes the agreement in the mechanical/fracture response and does not represent a quantitative error bound for the predicted damage field. Damage localization was evaluated separately, and the predicted high-damage regions showed good spatial agreement with the experimentally observed fracture locations.
3. The thread rolling simulation reveals that damage accumulation is mainly localized at the thread root and the transition region between the thread flank and root. The behavior is associated with the combined effects of geometric constraint, high equivalent plastic strain accumulation, and stress-state-dependent fracture strain. The thread-root transition therefore represents the most critical region for damage accumulation and numerical failure under the investigated rolling condition.

## Figures and Tables

**Table 1:** The chemical composition of the TC16 titanium alloy.

Element	Al	Mo	V	Fe	Si	C	O	N	H	Ti
wt%	3.00	5.28	4.40	0.18	<0.02	0.01	0.12	0.01	0.002	Bal.

**Table 2:** The mechanical test parameters.

Test Sample	Deformation Temperature	Strain Rate
Smooth tensile	20 °C	0.001, 0.01, 0.1, 1 s^−1^
Smooth tensile	100, 200, 300 °C	0.001 s^−1^
Six geometries (smooth, notched R = 3/R = 13, 0°/45°/90° shear)	20 °C	0.001 s^−1^

**Table 3:** The thread rolling model parameters.

Parameters		Value
Thread roller	Tooth tip radius *r*_1_	0.17 mm
	Tooth root radius *r*_2_	0.1 mm
	Medium diameter *D*_2_	148.85 mm
	Number of thread heads *N*	27
	Pitch *P*	1 mm
	Tooth crown height *h*_1_	0.25 mm
	Tooth height *h*_2_	0.32 mm
	Width *L*	30 mm
Blank	Diameter *d*_0_	5.41 mm

**Table 4:** The extracted experimental data at different strain rates.

Strain Rates (s^−1^)	σ_0.08_ (MPa)	σ_0.10_ (MPa)	σ_0.12_ (MPa)
0.001	1087	1101	1109
0.01	1102	1136	1141
0.1	1189	1206	1212
1	1230	1247	1253

**Table 5:** The extracted experimental data at different temperatures.

Temperature (℃)	σ_0.08_ (MPa)	σ_0.10_ (MPa)	σ_0.12_ (MPa)
100	1003	1012	1021
200	910	929	935
300	825	846	852

**Table 6:** The parameter values of the J-C constitutive model of TC16 titanium alloy.

*A* (MPa)	*B* (MPa)	*n*	*C*	*m*	${\dot{\varepsilon }}_{0}$ (s^−1^)	*T_r_* (*K*)	*T_m_* (*K*)
1025	384	0.42	0.0261	0.8027	0.001	293	1923

**Table 7:** The fracture strain and average stress triaxiality of different samples.

Sample	Fracture Displacement (mm)	Avg. Stress Triaxiality $\bar{\eta }$	Fracture Strain $\varepsilon_{f}$
Smooth tensile	3.68	0.33	0.175
Notched (R = 3 mm)	2.28	0.55	0.152
Notched (R = 13 mm)	2.03	0.49	0.158
0° shear	1.18	0.05	0.208
45° shear	1.13	0.21	0.189
90° shear	1.07	0.15	0.196

**Table 8:** Comparison of experimentally measured and numerically predicted fracture displacements.

Specimen	Experimental Fracture Displacement (mm)	Simulated Fracture Displacement (mm)	Relative Deviation (%)
Smooth tensile	3.68	3.40	7.61%
Notched R3	2.28	2.11	7.46%
Notched R13	2.03	1.88	7.39%
0° shear	1.18	1.09	7.63%
45° shear	1.13	1.05	7.08%
90° shear	1.07	0.99	7.48%

## Data Availability

The original contributions presented in this study are included in the article. Further inquiries can be directed to the corresponding author.
